# Fetal Blood Flow and Genetic Mutations in Conotruncal Congenital Heart Disease

**DOI:** 10.3390/jcdd8080090

**Published:** 2021-07-30

**Authors:** Laura A. Dyer, Sandra Rugonyi

**Affiliations:** 1 Department of Biology, University of Portland, Portland, OR 97203, USA; dyer@up.edu; 2 Department of Biomedical Engineering, Oregon Health & Science University, Portland, OR 97239, USA

**Keywords:** hemodynamics, cardiac malformations, flow-induced heart defects, VEGF, semaphorin signaling, cardiac neural crest cells, outflow tract

## Abstract

In congenital heart disease, the presence of structural defects affects blood flow in the heart and circulation. However, because the fetal circulation bypasses the lungs, fetuses with cyanotic heart defects can survive in utero but need prompt intervention to survive after birth. Tetralogy of Fallot and persistent truncus arteriosus are two of the most significant conotruncal heart defects. In both defects, blood access to the lungs is restricted or non-existent, and babies with these critical conditions need intervention right after birth. While there are known genetic mutations that lead to these critical heart defects, early perturbations in blood flow can independently lead to critical heart defects. In this paper, we start by comparing the fetal circulation with the neonatal and adult circulation, and reviewing how altered fetal blood flow can be used as a diagnostic tool to plan interventions. We then look at known factors that lead to tetralogy of Fallot and persistent truncus arteriosus: namely early perturbations in blood flow and mutations within VEGF-related pathways. The interplay between physical and genetic factors means that any one alteration can cause significant disruptions during development and underscore our need to better understand the effects of both blood flow and flow-responsive genes.

## 1. Introduction

**Figure 1:**
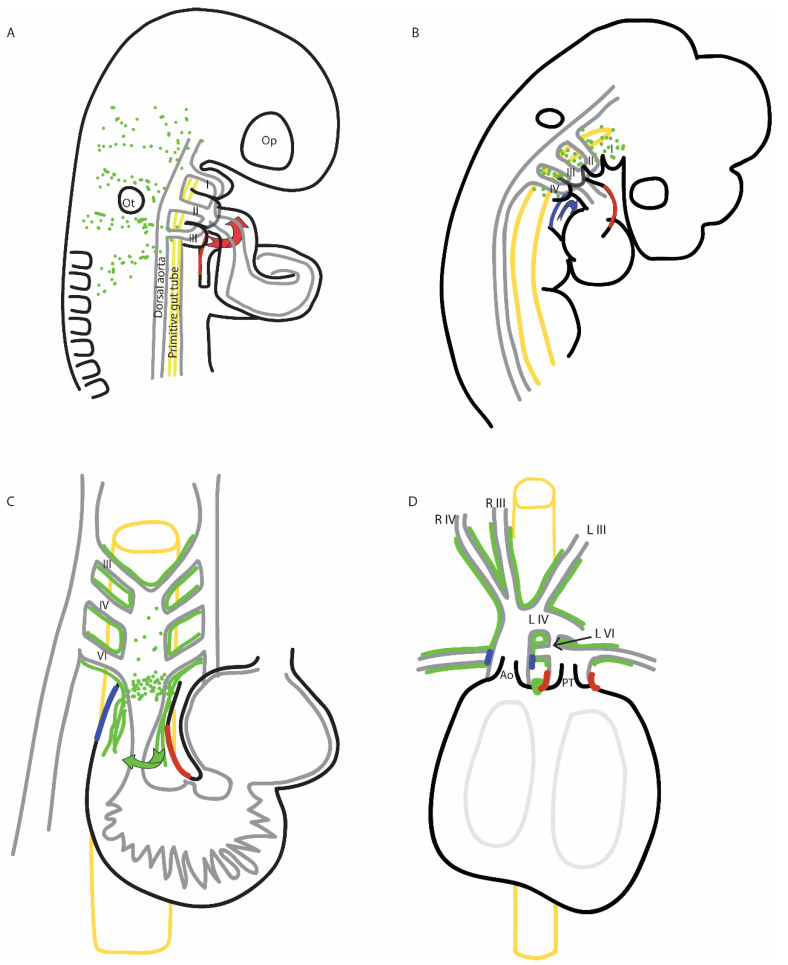
Schematics depict the cardiac neural crest (green) and the right side of the second heart field (red, blue) as the cells migrate toward the outflow tract, with stages based on the chick embryo. Mouse heart development is very similar, as vertebrate developmental processes are highly conserved. (**A**) At Hamburger-Hamilton (HH) stage 14, the heart is tubular, and the second heart field contributes myocardium (red) to the arterial pole; this myocardium spirals in, such that the right side of the second heart field will contribute to the left side of the outflow tract (see where the red cells migrate in panels (**B**–**D**). The cardiac neural crest cells (green) migrate in streams through the circumpharyngeal ridge. Blood exits the outflow tract through the second pharyngeal arch arteries. (**B**) By HH 18, the second heart field begins contributing smooth muscle (blue), and this addition does not spiral. The cardiac neural crest cells have arrived in the pharyngeal arches, labeled with Roman numerals. The outflow tract has moved caudally and is now in line with the third pharyngeal arch arteries. (**C**) By HH 26, the cardiac neural crest cells have surrounded the still-bilateral pharyngeal arch arteries and begun populating the outflow tract cushions, as depicted in this ventral view. As the cardiac neural crest cells populate the outflow tract cushions, they spiral in, causing additional rotation of the future aorta and pulmonary outlets. (**D**) By HH 32, the heart has four chambers and four forming valves (two semilunar valves and two atrioventricular valves), the outflow tract has been septated to form the base of the aorta (Ao) and pulmonary trunk (PT), and the pharyngeal arch arteries have remodeled. Blood flow between the aorta and pulmonary trunk is maintained via the ductus arteriosus (arrow). Pharyngeal arch artery derivatives are labeled based on side (L, left; R, right) and arch number (III, IV, or VI). Ao, aorta; PT, pulmonary trunk. The dorsal aortae and primitive gut tube are included for context. Op, optic vesicle; Ot, otic vesicle. Red, blue, and green arrows indicate the direction of migration.

During embryonic development, the heart is unique in that it must be functional prior to its maturation. Additionally, the heart is constructed in stages, becoming more complex as it develops and the needs of the embryo increase. As the heart forms, defects in some regions are more tolerable than others. The initial tubular heart, generated from the first heart field, is a simple two-layered contractile tube that promotes one-way flow. Defects that inhibit the formation or function of this heart tube are generally incompatible with life. As the heart becomes more complex with the addition of cells from the second heart field, the initially straight tubular heart elongates and loops, setting the stage for atria versus ventricles and the development of parallel flow (see Figure 1). The second heart field rotates during its migration into the outflow portion of the heart tube, which promotes the rotation of the outflow tract itself and correct alignment of the pulmonary trunk [1]. In mouse, this rotation has even been termed the “pulmonary push” as it nudges the pulmonary trunk into the correct orientation [2].

Blood flow exits the early heart tube through a single outflow tract, which is subsequently septated (divided) by the cardiac neural crest to generate the base of the great arteries: the pulmonary trunk and aorta [3]. As the cardiac neural crest cells populate the outflow tract cushions to septate the single outflow tract, the arteries that direct blood from the outflow tract undergo their own, concomitant asymmetric remodeling process. The outflow tract of the heart opens into the aortic sac, with blood exiting through three pairs of initially symmetrical pharyngeal arch arteries (PAAs). Asymmetrical remodeling converts the aortic sac and PAAs into the aortic arch (left PAA IV), the brachiocephalic trunk (right PAA IV), the pulmonary trunk and ductus arteriosus (left PAA VI), and the common carotid arteries (left and right PAA III). Two small pulmonary arteries arise from the bilateral sixth PAAs; otherwise, the right sixth PAA typically regresses as blood flow decreases within this artery [4]. Between outflow tract and pharyngeal arch artery remodeling, the arteries leaving the heart are reorganized to the aorta with its major branches and the pulmonary trunk (Figure 1). 

Between outflow tract elongation, rotation, and remodeling and PAA remodeling, there are extensive opportunities for abnormalities to arise. Rotation during migration of the second heart field establishes asymmetry, and abnormalities in the addition of the second heart field or this rotation lead to congenital heart defects, such as tetralogy of Fallot (TOF) [2,5]. One of the four defining characteristics of TOF is stenosis or complete atresia of the pulmonary outlet. Outflow tract septation failure results in a persistent singleton artery, known as persistent truncus arteriosus. Because hearts with either of these anomalies may present with a single great artery, diagnosis can be challenging [6]. One commonality, though, is that these defects are not necessarily fatal during fetal life due to the nature of fetal circulation.

## 2. Fetal Heart Circulation 

In the uterus, oxygenated blood is delivered to the fetus via the placenta, so the pulmonary circulation does not need to be isolated from the systemic circulation. In the neonatal and adult heart, the pulmonary and systemic circulations are separate. Deoxygenated blood comes from the body and enters the right atrium (RA). Blood in the RA is pumped to the right ventricle (RV), and the RV pumps blood into the pulmonary trunk so that blood can get oxygenated in the lungs. Oxygenated blood then returns to the heart via the left atrium (LA); is pumped to the left ventricle (LV); and exits via the aorta, which directs blood to the body.

**Figure 2:**
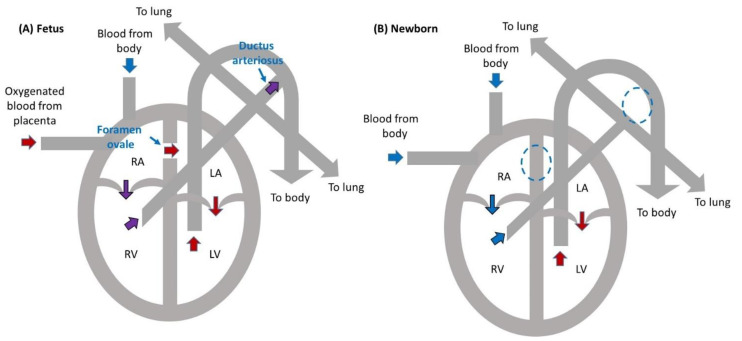
Heart circulation in a fetus and newborn baby. (**A**) Fetal circulation. The right and left atria are connected by the foramen ovale. The ductus arteriosus redirects blood from the pulmonary circulation to the systemic circulation. Oxygenated blood from the placenta is then directed to the left atrium (LA), through the left ventricle (LV), and to the systemic circulation. The lungs are bypassed. (**B**) Newborn circulation. The foramen ovale and ductus arteriosus close soon after birth (broken circles indicate fetal location), separating the right heart, which directs deoxygenated blood (in blue) from the body to the lungs for oxygenation; from the left heart, which pumps oxygenated blood (in red) from the lungs to the body. RA: right atrium; LA: left atrium; RV: right ventricle; LV: left ventricle.

While the fully formed fetal heart consists of two ventricles and two atria, like the adult and neonatal heart, the pulmonary and systemic circulations of the fetal heart are connected (see Figure 2). The RA and LA of the fetal heart are connected through the foramen ovale, which is present prior to closure of the atrial septum (the wall that separates the RA and LA) that occurs shortly after birth. In addition, the ductus arteriosus connects the pulmonary trunk to the aortic arch, allowing blood to co-mingle within both divisions of the circulatory system. Oxygenated blood comes from the placenta into the RA, where it is mostly directed to the LA through the foramen ovale. Further, blood in the RV is pumped to the pulmonary trunk but directed to the body circulation by the ductus arteriosus. Closure of the foramen ovale and ductus arteriosus soon after birth allow efficient oxygenation in the lungs and distribution of oxygenated blood to the body. Therefore, congenital heart defects that alter the aortic or pulmonary outlets, such as TOF and persistent truncus arteriosus, are not necessarily fatal during fetal life but can become deadly due to cyanosis (lack of proper blood oxygenation) right after birth.

**Figure 3:**
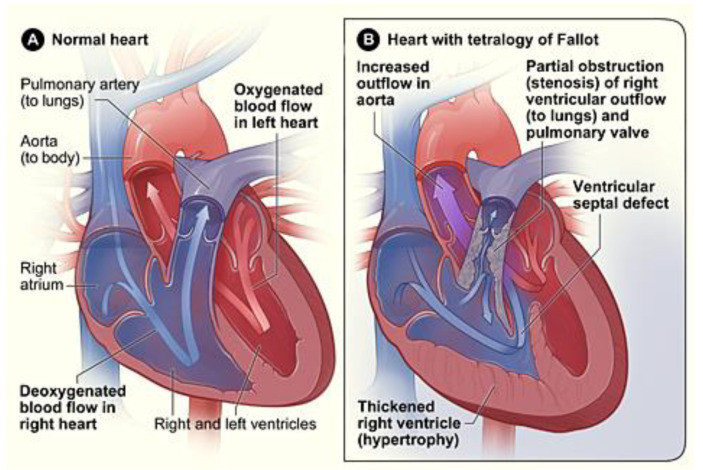
Blood flow within a normal and malformed heart after birth. (**A**) Cardiac structure and blood flow in a normal heart. Deoxygenated blood in the right of the heart, shown in blue, is completely separated from the oxygenated blood in the left heart, shown in red. Deoxygenated blood is pumped into the lungs through the pulmonary artery to get oxygenated. Oxygenated blood returns to the left heart and is pumped to the body through the aorta. (**B**) Cardiac structure and blood flow in a heart with Tetralogy of Fallot (TOF), a cyanotic congenital heart defect. Unlike the normal heart, the TOF heart exhibits a ventricular septal defect (VSD) that allows oxygenated and deoxygenated blood to mix; stenosis of the pulmonary trunk or valve that prevents or diminishes blood flow to the lungs, as well as a thickened right ventricular wall and a change in the normal positioning of the aorta relative to the pulmonary trunk, such that the aorta sits on top of the VSD. Reproduced from the public domain in Wikimedia Commons (https://commons.wikimedia.org/wiki/File:Tetralogy_fallot.jpg, accessed on 22 July 2021), image source: National Institutes of Health (NIH).

Among cyanotic heart defects, TOF is perhaps one of the most frequently encountered. Compared with the normal circulation, deoxygenated and oxygenated blood are mixed and flow to the lungs is impaired in the TOF heart (see Figure 3). TOF is characterized by four defects: (i) an outlet ventricular septal defect (VSD), due to anterior malalignment of the outflow septum that separates the RV and LV, and allows mixing of oxygenated and deoxygenated blood; (ii) stenosis or narrowing of the subpulmonary outflow tract, impairing blood flow to the lungs; (iii) an over-riding or dextrapositioned aorta; and (iv) a hypertrophic RV, which manifests as a thickened RV wall. A baby born with a TOF defect soon becomes cyanotic, and surgical intervention is frequently required right away. To gain time for planning, doctors can keep the ductus arteriosus open (patent) by administering prostaglandin, so more oxygenated blood reaches the body circulation. Fetal interventions to increase blood flow to the lungs before it is critically needed are also possible. Fetal diagnosis of congenital heart disease is important for proper planning before birth, leading to better outcomes especially for babies with cyanotic heart defects.

## 3. Abnormal Blood Flow in Congenital Heart Disease

The prenatal diagnosis of complex congenital heart disease (CHD) is becoming more common than postnatal diagnosis. Diagnosis with ultrasound techniques can be done as early as 10 weeks of gestation, although 11–13 weeks is indicated for women with familial history of CHD, previous child with CHD, or early findings associated with CHD [7]. For reference, the human heart is fully formed at week 8 of gestation, although cardiac muscle cells are not fully organized until week 20 [8]. The earlier the ultrasound scan, the less precise the diagnosis because more features can be observed and measured at later gestation stages as the heart becomes bigger [7]. Early diagnosis, however, creates an opportunity for early intervention and planning to improve outcomes [9]. For example, a recent study [10] found that in babies with TOF, fetal echocardiography performed around 25 weeks of gestation was predictive of ductal dependence on neonates, who will then require surgical intervention soon after they are born [10]. In particular, the study found that echocardiography measurements of the pulmonary valve and main pulmonary artery diameters, together with the direction of flow in the ductus arteriosus, predict ductal dependence [10]. Related studies found that prenatal diagnosis of CHD was associated with decreased neonatal morbidities [11] and emergency surgery [9] relative to infants with postnatal diagnoses, and that babies with a prenatal diagnosis of critical CHD were significantly less likely to die prior to cardiac surgery than those with a comparable postnatal diagnosis [12]. These improvements are possible because prediction of outcomes from fetal diagnosis, when there is plenty of time for surgical strategy development, can help plan for proper neonatal interventions, including their optimal timing, saving time when the baby is born.

**Figure 4:**
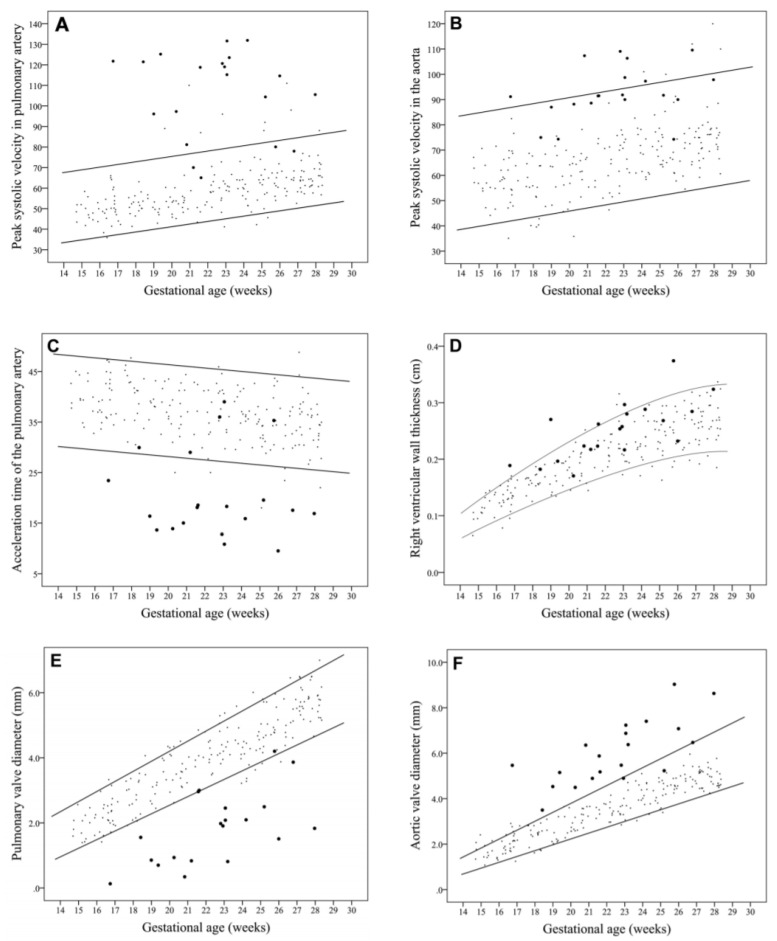
Comparison of absolute values of cardiac parameters measured using echocardiography at various gestational ages among healthy fetuses (small dots) and fetuses with TOF (large dots). Lines represent the 5th and 95th percentiles of normal reference ranges. (**A**) Peak systolic velocity in pulmonary artery; (**B**) Peak systolic velocity in the aorta; (**C**) Acceleration time of the pulmonary artery; (**D**) Right ventricular wall thickness; (**E**) Pulmonary valve diameter; (**F**) Aortic valve diameter. Reproduced with permission from [13].

Babies with TOF, particularly those with neonatal ductal dependence, benefit from prenatal diagnosis: prostaglandin administration, which keeps the ductus arteriosus open, can be anticipated, avoiding hypoxic brain damage [13], and surgeries can be carefully timed and planned. To determine which cardiac functional parameters can be employed for diagnosis using echocardiography methods, a recent study [13] compared data from 20 TOF fetal echocardiographies versus 200 healthy fetal heart echocardiographies ranging from 11 to 28 weeks of gestation [13]. They found that peak systolic velocity in both the pulmonary artery and aorta were elevated in fetuses with TOF, and while pulmonary valve diameter was reduced, aortic valve diameter was increased in TOF hearts relative to the control hearts (see Figure 4). Therefore, a combination of measurements can be used to diagnose and confirm the diagnosis of TOF specifically, and congenital heart disease in general, in unborn babies.

A recent study [14] analyzed the dynamics of blood flow in 3 fetal hearts with TOF and compared them with normal fetal hearts. The study used a combination of echocardiography and computational fluid dynamics (CFD) to decipher differences in how blood flows in the fetal TOF hearts in comparison to normal cardiac flow. While the TOF hearts presented structural differences among themselves and with respect to normal hearts, the study generally found that right to left shunting in the ventricles (that is, flow that is diverted from the RV to the LV through the VSD) depended on the size of the VSD. Further, the dynamics of blood flow in the vicinity of the outflow and VSD depended on the degree of overriding aorta (i.e., the position of the aorta with respect to the VSD). In the same study, echocardiography data showed that the stroke volume was elevated in hearts with TOF. This finding is consistent with data from [13], which measured elevated velocities in hearts with TOF. CFD simulations further showed that systolic LV work is elevated in hearts with TOF and that there is pressure equilibration between the RV and LV due to the VSD. Furthermore, the dynamics of blood flow in the RV exerted elevated wall shear stress on RV walls, and the endocardium surrounding the VSD was also exposed to elevated wall shear stress. Because wall shear stress is an important inducer of remodeling through mechanotransduction mechanisms, this elevated wall shear stress could have implications for fetal cardiac wall remodeling. 

## 4. Early Perturbed Flow and TOF

Blood flow dynamics (hemodynamics) affect the development of the heart [15,16] and aortic arches [17,18,19] leading to congenital heart defects. As the heart and vasculature develop, they are very sensitive to blood flow conditions. Diverse mechanosensitive and mechanotransduction mechanisms translate mechanical inputs from blood flow into signaling cascades that in turn affect further development, including growth and remodeling of cardiovascular tissues. These mechanotransduction mechanisms therefore act as a feedback loop, enabling the heart and vasculature to develop in tune with the fast-evolving embryo needs. These mechanisms allow the embryo to adapt and survive changing conditions, including environmental and maternal effects. The downside is that when blood flow in the embryo is abnormal, for example due to maternal diabetes [20] or alcohol consumption [21], these adaptations can be compounding and lead to heart malformations that are independent of genetic anomalies in the embryo.

Hemodynamics can alter heart development independent of genetic or toxic exposures. Blood flow affects the expression of mechanosensitive genes and regulation of proteins [22,23,24,25,26], leading to abnormal valve formation [27,28] and heart defects, including TOF [16]. Despite extensive ongoing studies, the molecular mechanisms behind mechanosensitivity and mechanotransduction are not yet fully understood, in part due to the complexity of the signaling networks involved. Nevertheless, altered blood flow is an important, and yet frequently forgotten factor when looking at causes of congenital heart defects: not only can perturbed flow alone lead to heart defect, but the presence of a structural heart defect will in turn alter blood flow, perpetuating a cycle of maladaptation.

## 5. Genetic Mutations Associated with TOF

As mentioned above, TOF is characterized by four distinct cardiac defects: subpulmonary outlet stenosis, right ventricular hypertrophy, overriding or dextrapositioned aorta, and outlet ventricular septal defects. Stenosis of the subpulmonary outlet can be caused by reduced contributions from the second heart field during outflow tract elongation and rotation. In cases of complete pulmonary atresia, the single outflow vessel can be identified as the aorta based on the diameter of the vessel, the presence of three semilunar valve leaflets, and placement of the coronary arteries [29]. Interestingly, patients with TOF often have a PAA remodeling anomaly as well, typically involving regression of the left fourth PAA [30].

In persistent truncus arteriosus, the cardiac neural crest fails to populate the outflow tract cushions, which are then unable to septate the outflow tract into the aorta and pulmonary trunk. The single outflow vessel is larger than the aorta of hearts with pulmonary atresia, and the coronary arteries are oriented on the aortic portion of the common trunk. Additionally, the valve associated with this single vessel has as many as six semilunar valve leaflets. Because the cardiac neural crest migrates through the pharyngeal arches and induces remodeling of the pharyngeal arch arteries, the co-occurrence of PAA defects is unsurprising. Although the PAA anomalies vary, persistent truncus arteriosus is more commonly associated with an absent right fourth PAA and bilateral regression of the sixth PAAs [31,32]. Given the similar etiologies of TOF and persistent truncus arteriosus and their different associated PAA anomalies [30,31], we will focus on genetic causes of these two defects, with a particular interest in blood flow-responsive signaling pathways.

### 5.1. The Vascular Endothelial Growth Factor (VEGF) Family

**Figure 5:**
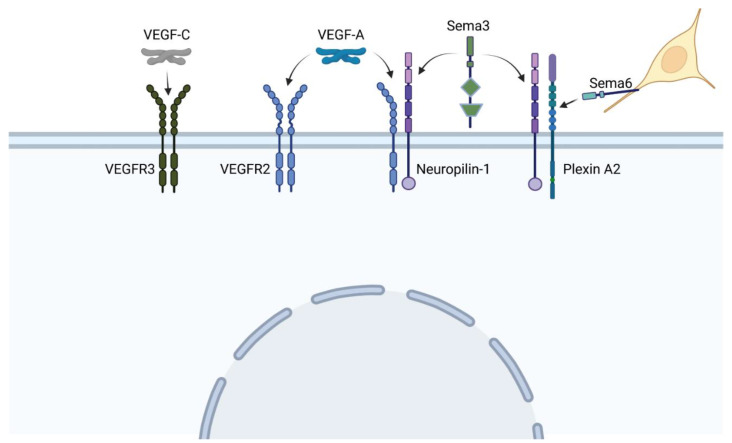
VEGF and sempahorin (Sema) ligands bind to a variety of heterodimer receptors, and co-receptor Neuropilin-1 can heterodimerize with both VEGF and plexin receptors, leading to extensive cross-talk between pathways. Neural crest cells (yellow) express the membrane-bound semaphorin 6, which directs their migration toward specific plexin receptors. (Created with BioRender).

The vascular endothelial growth factor (VEGF) pathway is one of the most critical pathways for vascular development. In brief, secreted VEGF ligands bind to transmembrane VEGF receptors, which heterodimerize or homodimerize and autophosphorylate upon ligand binding (see Figure 5). Among the VEGF proteins, VEGF-A is the major family member associated with blood vessel formation and can generate numerous isoforms via alternative splicing. Among the 16 isoforms, VEGF-A_165_ was the first identified and remains the prototypical pro-angiogenic isoform (reviewed in [33]). VEGF receptor 2 (VEGFR2) can further signal independent of VEGF-A in the presence of high wall shear stress [34]. Adding more complexity to this pathway, VEGF receptors may heterodimerize with co-receptor neuropilin-1, which can also form heterodimers with the plexin receptors of the semaphorin-plexin signaling axis (Figure 5). Neuropilin-1 thus serves as a molecular switch between pro-angiogenic VEGF-A signaling and anti-angiogenic semaphorin signaling. Because vascular development relies on the level of VEGF-A signaling, mutations in any component of the VEGF-A or semaphorin pathways may lead to phenotypic anomalies. We will focus on mutations within these pathways that lead to TOF (summarized in Table 1) versus persistent truncus arteriosus (summarized in Table 2) and/or are associated with perturbations in blood flow. 

### 5.2. Mutations within the VEGF-A Pathway Are Associated with TOF

Patients with TOF and mutations in the VEGF pathway have an increased risk of presenting with an absent pulmonary valve and a right-sided aortic arch compared with patients with TOF who have other genetic variations [35]. Single nucleotide polymorphisms (SNPs) within VEGF-A that have been associated with TOF include -C2578A (rs699947), -C634G (rs2010963), and C936T (rs3025039). These specific SNPs are within the promoter or untranslated regions of the VEGF-A gene and reduce VEGF-A expression, thus increasing the risk of developing TOF [36]. Intriguingly, an estrogen receptor within the VEGF-A promoter limits the effects of the C936T substitution among females and reduces their risk of developing TOF [37,38]. 

Genetic manipulation of the VEGF-A expression level in mouse supports the importance of VEGF-A during development. When the first VEGF-A knockout mice were generated, even lacking one copy of this gene resulted in mid-gestational lethality due to abnormal yolk sac vasculogenesis, impaired hematopoiesis, and stunted heart development [39,40]. A hypomorphic lacZ-knock-in VEGF-A allele allowed for the survival of fertile heterozygous mice, but the homozygous hypomorphs still died by embryonic day 9.0 and show the same impaired hematopoiesis and vasculogenesis as observed in the original VEGF-A heterozygotes [41]. 

VEGF receptor 2 (VEGFR2, also known as KDR) is broadly expressed in the vascular endothelium and blood islands and is critical for their development [21,22,23,24]. The global VEGFR2 knockout is embryonic lethal between embryonic days 8.5 and 9.5 due to failure to form the blood islands or blood vessels [42]. At later stages, this receptor is maintained in the endothelium of the pharyngeal arch arteries, where it is phosphorylated in response to blood flow. Although both the left and right sixth PAAs express VEGFR2, reduced blood flow through the right sixth arch artery means that VEGF signaling is absent; thus, only the left sixth PAA, with its active VEGF signaling, is maintained and remodeled to give rise to the ductus arteriosus [43]. Although PAAs III and IV express VEGF receptor 2 [43], they do not regress if VEGFR2 phosphorylation is inhibited [44]. This difference may be related to differential neural patterning through the arches, though the molecular mechanisms remain unclear (reviewed in [45]). Stop-gain and deleterious missense mutations in VEGFR2 have been identified in patients with TOF, particularly those with absent pulmonary valve or complete pulmonary atresia [35]. These phenotypes are consistent with VEGFR2’s critical role in maintaining the left sixth PAA.

Unexpectedly, mutations within VEGF receptor FLT4 (i.e., VEGFR3) can also lead to TOF. VEGFR3 is initially expressed in small mesenchymal clusters within the pharyngeal arches (embryonic day 9.5–10.5) and becomes restricted to Prox1-positive lymphatic cells during outflow tract septation [46]. VEGFR3 is typically associated with lymphangiogenesis because it promotes lymphatic endothelial cell migration upon binding to VEGF-C [47]. This receptor is indispensable for lymphatic vessels, and mutations are associated with lymphatic disorders. However, loss-of-function stop codons and nonsense mutations are associated with TOF and are distinct from the mutations that lead to lymphatic disorders [48]. VEGFR3′s restricted expression pattern, particularly because it is not expressed in the outflow tract endothelium [46], makes it challenging to visualize how truncation mutations specifically lead to TOF. Additionally, knockout mouse models exhibit cardiac failure and embryonic lethality by embryonic day 10.5 [49], preventing an analysis of how loss of VEGFR3 relates to the specific development of TOF.

### 5.3. Mutations within the VEGF-Semaphorin Crosstalk Mediator Neuropilin-1 Can Lead to TOF or Persistent Truncus Arteriosus

Among VEGF’s roles, low levels of VEGF promote the endothelial expression of Semaphorin 3A, which then recruits neuropilin-1-expressing monocytes that stabilize developing blood vessels [50]. In contrast, high levels of VEGF inhibit vascular stability but are needed to initiate angiogenesis [50]. These reciprocal inhibitions suggest that the VEGF and semaphorin signaling pathways have distinct, critical roles during angiogenesis. Both ligands can bind to co-receptor neuropilin-1, which thus plays a key role in switching from VEGF to semaphorin signaling. Neuropilin-1 is a single-pass transmembrane protein with five distinct extracellular domains. Two domains (a1 and a2) facilitate semaphorin binding; two other domains (b1 and b2) facilitate VEGF-A_165_ binding. The final extracellular domain is the MAM domain, which may support—but does not directly cause—dimerization with other receptors [51]. This MAM domain is the major distinction between endothelial-specific, full-length neuropilin-1 and soluble neuropilin-1 isoforms that lack this domain and are not expressed in the endothelium. 

Full-length neuropilin-1 enhances VEGF-A_165_ binding to VEGFR2 and promotes cytoskeletal remodeling [52]. When neuropilin-1 is conditionally knocked out of endothelial cells, the resulting mice exhibit a single outflow vessel. Although neuropilin-1 can bind to both semaphorin and VEGF, the VEGF binding appears key for its role in outflow tract septation. Knocking in a neuropilin-1 mutation that eliminates the semaphorin-binding site yields normal outflow tract septation [53].

Two neuropilin-1 mutations are associated with TOF. V733I occurs within the MAM domain and is predicted to yield a nonsense mutation [54,55]. Neuropilin-1 splice variants that lack the MAM domain lead to soluble isoforms that act as decoy receptors to downregulate VEGF signaling [56]. These soluble neuropilin splice variants specifically sequester VEGF-A_165_, thus inhibiting VEGF-A’s functions and leading to increased endothelial apoptosis, decreased blood vessel numbers, and decreased vascular density [57,58]. Intriguingly, VEGF-A induces a proteolytic cleavage of the extracellular binding domains (a1–b2) of neuropilin-1, creating an additional soluble neuropilin-1 decoy that downregulates both VEGF and semaphorin signaling, creating a negative feedback loop [52,59].

The other TOF-associated neuropilin-1 mutation is within the 3′ UTR (rs10080) and is predicted to be a miRNA-binding site [55]. A patient presented with TOF with double aortic arch and a 1.8 Mb deletion on chromosome 11 that reduced their expression level of neuropilin-1 [59]. Neuropilin-1 can repress VEGF-A signaling when neuropilin-1 and VEGFR2 are expressed on adjacent cells, forming *trans* heterodimers [60]; thus, the neuropilin-1 expression is likely key for normal vascular development. 

In contrast to the neuropilin-1 variants that lack the MAM domain and supporting neuropilin-1′s role in mediating a switch between VEGF and semaphorin signaling, a splice site mutation that leads to a truncated neuropilin-1 has been linked to persistent truncus arteriosus [61]. In the patient with this truncated neuropilin-1, the truncation prevents the translation of any functional protein. This patient data is supported by the neuropilin-1 knockout mouse, which presents with regression of left PAA IV and VI and results in persistent truncus arteriosus [62].

Adding additional complexity to the neuropilin-VEGF pathway, whether neuropilin-1 and VEGFR2 form *cis* heterodimers within a single cell or *trans* heterodimers when expressed in different cells determines which downstream ERK is phosphorylated. Activation of the *trans* heterodimers specifically phosphorylates the kinase ERK2, compared with *cis* heterodimers leading to ERK1 phosphorylation [60]. Of these two kinases, ERK2 is located near the classical 22q11.2 deletion, and haploinsufficiency of ERK2 is associated with persistent truncus arteriosus and right-sided aortic arch [63].

### 5.4. Mutations within the Semaphorin Signaling Pathway Lead to TOF or Persistent Truncus Arteriosus

The canonical semaphorin signaling pathway consists of the semaphorin ligands, which may be secreted (e.g., class III) or transmembrane (e.g., class VI) proteins, and their plexin receptors, which fall into four major classes (A–D). As with the VEGF pathway, the receptors form heterodimers or homodimers, and heterodimers can be formed with neuropilin 1 [64]. The downstream effects of each pathway depend on the affinity of the semaphorin ligands that are present for the receptors that are expressed, and many of these ligand-receptor combinations impact vascular development.

Among the class III semaphorins, semaphorin 3A is flow-sensitive, and its expression is enriched in the anti-atherosclerotic portion of the adult aortic arch and is downregulated by oscillatory flow [65]. Additionally, it is the semaphorin ligand with the highest binding affinity for neuropilin-1 [56]. When VEGF-A levels decrease, semaphorin 3A helps recruit neuropilin 1-expressing monocytes to stabilize blood vessels [50]. Semaphorin 3A competitively inhibits VEGF-A_165_ from binding to neuropilin-1-VEGFR2 heterodimers and can block VEGF-A_165_ and VEGF-A_121_ activity in a neuropilin-1-indepdendent manner [66]. Thus, mutations in semaphorin 3A may lead to aberrantly high levels of VEGF signaling or the inability to halt VEGF-induced chemotaxis, causing over-remodeling at the expense of vascular stabilization. Perhaps unsurprisingly, semaphorin 3A mutations and copy number variations in semaphorin 3D and 3E are associated specifically with TOF [67]. Vascular instability may help explain the pulmonary stenosis or atresia observed in TOF.

Semaphorin 3C plays a key role in directing cardiac neural crest cells to the outflow tract. It is expressed in the second heart field mesoderm and repressed in the neural crest cells as the second heart field is adding to the outflow tract and the cardiac neural crest cells have reached the pharyngeal arches [68]. As the cardiac neural crest migrate into the outflow tract, semaphorin 3C is then observed in the myocardial cuff surrounding the outflow tract and the cardiac neural crest themselves [69,70]. Because semaphorin 3C causes aggregation of the cardiac neural crest [68], these expression patterns suggest that semaphorin 3C is key for outflow tract septation. Receptors plexin D1 and neuropilin-1 are present in the endothelium of the aortic arch [70]. In the semaphorin 3C-null mouse embryo, the cardiac neural crest fails to migrate into the outflow tract [71], and semaphorin 3C-null mice exhibit persistent truncus arteriosus and interrupted aortic arch types B and C [72].

Unique among the semaphorins, semaphorin 3E can bind to plexin D1 in the presence [73] or absence [74] of neuropilin. Thus, this ligand may serve as another switch between plexin D1-associated persistent truncus arteriosus and plexin A2-associated TOF. When bound to plexin D1, semaphorin 3E inhibits endothelial migration and tube formation by destabilizing the actin cytoskeleton [75]. Mutations in semaphorins 3E and 4A, along with receptor plexin D1, are associated with persistent truncus arteriosus [76,77], which suggests that these guidance cues are critical for neural crest migration during arterial pole septation. However, when semaphorin 3E binds plexin D1, it can block VEGF-induced Akt phosphorylation to inhibit endothelial migration and tube formation [78]. Insufficiency for semaphorin 3E would thus allow excessive endothelial migration, suggesting a mechanism for pulmonary stenosis or atresia. In support of this hypothesis, some patients with TOF have copy number loss of chromosome 7q21.11, which contains semaphorin 3D and semaphorin 3E [67].

Intriguingly, though, plexin D1 is a mechanosensor that can form a complex with neuropilin-1 and VEGFR2 and can act independently of semaphorin 3E to induce a response to wall shear stress [79]. This force-transduction role opens new questions about how plexin D1 and its semaphorin ligands contribute to outflow tract and pharyngeal arch artery remodeling in response to blood flow.

Despite including both class III secreted (3A, 3C, and 3F) and class VI transmembrane (6A and 6B) semaphorin ligands, all five of these ligands can bind to receptor plexin A2 [70,80,81]. Plexin A2 is expressed by cardiac neural crest cells and is observed in the mesenchyme of the pharyngeal arches and the outflow tract during cardiac neural crest migration toward the outflow tract [70,71]. During outflow tract septation, plexin A2 is particularly enriched where the neural crest prongs are fusing together [69]. Cardiac neural crest cells express plexin A2 and are thus attracted to semaphorin 3C, which is observed in the outflow tract myocardium, but repulsed from cells that express transmembrane semaphorin 6A or 6B. Semaphorin 6A and 6B are predominantly expressed in the dorsal neural tube and somites during neural crest migration and may thus help direct the neural crest to migrate ventrally. Consistently, the plexin A2-null mouse displays persistent truncus arteriosus and two variations of interrupted aortic arch [70]. However, these phenotypes show variable penetrance, underscoring the complexity of compensation. Highlighting this complexity, loss of plexin A2 copy number has been identified in patients with TOF [67]. 

## 6. Conclusions

Perturbed cardiac fetal blood flow and congenital heart disease are intrinsically interlinked. The presence of subtle or overt structural heart defects induces blood flow conditions that differ from normal. Because blood flow provides important mechanical feedback during heart development, mutations in genes that are responsive to flow (such as VEGF and semaphorin) or perturbations in blood flow itself both lead to congenital heart defects. The abnormal changes in blood flow in congenital heart disease then perpetuate a vicious cardiovascular remodeling cycle. Advances in genomic sequencing are allowing the detection of ultra-rare genomic variants, such as Notch1 missense mutations [82], and studies in zebrafish have begun linking blood flow alterations to Notch1 signaling and TOF [83]. The plethora of flow-related genes and the numerous possible mutations make prenatal genetic diagnosis of a congenital heart defect challenging. However, the perturbations in blood flow can be used to our advantage to diagnose the severity of heart defects as well as ductal dependence during fetal stages, allowing more time for planning interventions after the baby is born. Several of the genetic mutations frequently associated with TOF and persistent truncus arteriosus, our focus of discussion in this paper, are associated with flow-responsive VEGF signaling pathways. This association underscores the importance of blood flow and blood flow feedback mechanisms on the development of the heart. 

## Figures and Tables

**Table 1:** Mutations associated with tetralogy of Fallot.

Gene	Mutation	Mutation Effects, If Known
VEGF-A	C2578A C634G C936T Haploinsufficiency (mouse)	Reduces VEGF-A expression Reduces VEGF-A expression Reduces VEGF-A expression Abnormal vasculogenesis and hematopoiesis
VEGFR2	Stop-gain Missense Knockout (mouse)	Loss of blood islands
VEGFR3	Loss-of-function Nonsense	
Neuropilin-1	V733I Predicted miRNA binding site mutation	Sequesters VEGF-A Reduces neuropilin-1 expression
PlexinA2	Copy number loss	
Semaphorin 3D	Copy number loss	
Semaphorin 3E	Copy number loss	Destabilizes actin cytoskeleton

**Table 2:** Mutations associated with persistent truncus arteriosus.

**Gene**	**Mutation**	**Mutation Effects, If Known**
Erk2	Haploinsufficiency	
Semaphorin 3C	Null (mouse)	Reduces cardiac neural crest migration
Plexin A2	Null (mouse)	
Neuropilin-1	Truncation	No functional protein

## References

[1] Ward C., Stadt H., Hutson M., Kirby M.L. (2005). Ablation of the secondary heart field leads to tetralogy of Fallot and pulmonary atresia. Dev. Biol..

[2] Scherptong R.W., Jongbloed M.R., Wisse L.J., Vicente-Steijn R., Bartelings M.M., Poelmann R.E., Schalij M.J., Groot A.C.G.-D. (2012). Morphogenesis of outflow tract rotation during cardiac development: The pulmonary push concept. Dev. Dyn..

[3] Kirby M.L., Gale T.F., Stewart D.E. (1983). Neural crest cells contribute to normal aorticopulmonary septation. Science.

[4] Hiruma T., Hirakow R. (1995). Formation of the pharyngeal arch arteries in the chick embryo. Observations of corrosion casts by scanning electron microscopy. Anat. Embryol..

[5] Bertrand N., Roux M., Ryckebüsch L., Niederreither K., Dollé P., Moon A., Capecchi M., Zaffran S. (2011). Hox genes define distinct progenitor sub-domains within the second heart field. Dev. Biol..

[6] Kirby M.L. (2008). Pulmonary Atresia or Persistent Truncus Arteriosus. Circ. Res..

[7] Hernandez-Andrade E., Patwardhan M., Cruz-Lemini M., Luewan S. (2017). Early Evaluation of the Fetal Heart. Fetal Diagn. Ther..

[8] Pervolaraki E., Anderson R.A., Benson A.P., Hayes-Gill B., Holden A.V., Moore B.J.R., Paley M.N., Zhang H. (2013). Antenatal architecture and activity of the human heart. Interface Focus.

[9] Levey A., Glickstein J.S., Kleinman C.S., Levasseur S.M., Chen J., Gersony W.M., Williams I.A. (2010). The Impact of Prenatal Diagnosis of Complex Congenital Heart Disease on Neonatal Outcomes. Pediatr. Cardiol..

[10] Arya B., Levasseur S.M., Woldu K., Glickstein J.S., Andrews H.F., Williams I.A. (2013). Fetal Echocardiographic Measurements and the Need for Neonatal Surgical Intervention in Tetralogy of Fallot. Pediatr. Cardiol..

[11] Landis B.J., Levey A., Levasseur S.M., Glickstein J.S., Kleinman C.S., Simpson L.L., Williams I.A. (2012). Prenatal Diagnosis of Congenital Heart Disease and Birth Outcomes. Pediatr. Cardiol..

[12] Holland B.J., Myers J.A., Woods C.R. (2015). Prenatal diagnosis of critical congenital heart disease reduces risk of death from cardiovascular compromise prior to planned neonatal cardiac surgery: A meta-analysis. Ultrasound Obstet. Gynecol..

[13] Jatavan P., Tongprasert F., Srisupundit K., Luewan S., Traisrisilp K., Tongsong T. (2016). Quantitative Cardiac Assessment in Fetal Tetralogy of Fallot. J. Ultrasound Med..

[14] Wiputra H. (2018). Right Ventricular Physiology in Health and Disease. Am. J. Physiol. Heart Circ. Physiol..

[15] Emidgett M., Erugonyi S. (2014). Congenital heart malformations induced by hemodynamic altering surgical interventions. Front. Physiol..

[16] Midgett M., Thornburg K.L., Rugonyi S. (2017). Blood flow patterns underlie developmental heart defects. Am. J. Physiol. Circ. Physiol..

[17] Karakaya C., Goktas S., Celik M., Kowalski W.J., Keller B.B., Pekkan K. (2018). Asymmetry in Mechanosensitive Gene Expression during Aortic Arch Morphogenesis. Sci. Rep..

[18] Lashkarinia S.S., Çoban G., Ermek E., Çelik M., Pekkan K. (2020). Spatiotemporal remodeling of embryonic aortic arch: Stress distribution, microstructure, and vascular growth in silico. Biomech. Model. Mechanobiol..

[19] Celik M., Goktas S., Karakaya C., Cakiroglu A.I., Karahuseyinoglu S., Lashkarinia S.S., Ermek E., Pekkan K. (2020). Microstructure of early embryonic aortic arch and its reversibility following mechanically altered hemodynamic load release. Am. J. Physiol. Circ. Physiol..

[20] Lawson T.B., Scott-Drechsel D.E., Chivukula V.K., Rugonyi S., Thornburg K.L., Hinds M.T. (2018). Hyperglycemia Alters the Structure and Hemodynamics of the Developing Embryonic Heart. J. Cardiovasc. Dev. Dis..

[21] Karunamuni G., Gu S., Doughman Y.Q., Peterson L.M., Mai K., McHale Q., Jenkins M.W., Linask K.K., Rollins A.M., Watanabe M. (2014). Ethanol exposure alters early cardiac function in the looping heart: A mechanism for congenital heart defects?. Am. J. Physiol. Circ. Physiol..

[22] Groenendijk B.C., Hierck B.P., Groot A.C.G.-D., Poelmann R.E. (2004). Development-related changes in the expression of shear stress responsive genesKLF-2, ET-1, and NOS-3 in the developing cardiovascular system of chicken embryos. Dev. Dyn..

[23] Alser M., Shurbaji S., Yalcin H. (2021). Mechanosensitive Pathways in Heart Development: Findings from Chick Embryo Studies. J. Cardiovasc. Dev. Dis..

[24] Rasouli S.J., El-Brolosy M., Tsedeke A.T., Bensimon-Brito A., Ghanbari P., Maischein H.-M., Kuenne C., Stainier D.Y. (2018). The flow responsive transcription factor Klf2 is required for myocardial wall integrity by modulating Fgf signaling. Elife.

[25] Midgett M., López C.S., David L., Maloyan A., Rugonyi S. (2017). Increased Hemodynamic Load in Early Embryonic Stages Alters Endocardial to Mesenchymal Transition. Front. Physiol..

[26] Midgett M., López C.S., David L., Maloyan A., Rugonyi S. (2017). Increased Hemodynamic Load in Early Embryonic Stages Alters Myofibril and Mitochondrial Organization in the Myocardium. Front. Physiol..

[27] Menon V., Eberth J.F., Goodwin R.L., Potts J.D. (2015). Altered Hemodynamics in the Embryonic Heart Affects Outflow Valve Development. J. Cardiovasc. Dev. Dis..

[28] Steed E., Faggianelli N., Roth S., Ramspacher C., Concordet J.-P., Vermot J. (2016). klf2a couples mechanotransduction and zebrafish valve morphogenesis through fibronectin synthesis. Nat. Commun..

[29] Dyer L.A., Kirby M.L. (2009). Sonic hedgehog maintains proliferation in secondary heart field progenitors and is required for normal arterial pole formation. Dev. Biol..

[30] Hanneman K., Newman B., Chan F. (2017). Congenital Variants and Anomalies of the Aortic Arch. Radiographics.

[31] Nishibatake M., Kirby M.L., Van Mierop L.H. (1987). Pathogenesis of persistent truncus arteriosus and dextroposed aorta in the chick embryo after neural crest ablation. Circulation.

[32] Tomita H., Connuck D.M., Leatherbury L., Kirby M.L. (1991). Relation of Early Hemodynamic Changes to Final Cardiac Phenotpe and Survival After Neural Crest Ablation in Chick Embryos. Circulation.

[33] Peach C.J., Mignone V.W., Arruda M.A., Alcobia D.C., Hill S.J., Kilpatrick L.E., Woolard J. (2018). Molecular Pharmacology of VEGF-A Isoforms: Binding and Signalling at VEGFR2. Int. J. Mol. Sci..

[34] Vion A.-C., Perovic T., Petit C., Hollfinger I., Bartels-Klein E., Frampton E., Gordon E., Claesson-Welsh L., Gerhardt H. (2021). Endothelial Cell Orientation and Polarity Are Controlled by Shear Stress and VEGF Through Distinct Signaling Pathways. Front. Physiol..

[35] Reuter M.S., Jobling R., Chaturvedi R.R., Manshaei R., Costain G., Heung T., Curtis M., Hosseini S.M., Liston E., Lowther C. (2018). Haploinsufficiency of vascular endothelial growth factor related signaling genes is associated with tetralogy of Fallot. Genet. Med..

[36] Lambrechts D., Devriendt K., Driscoll D.A., Goldmuntz E., Gewillig M., Vlietinck R., Collen D., Carmeliet P. (2005). Low expression VEGF haplotype increases the risk for tetralogy of Fallot: A family based association study. J. Med Genet..

[37] Li X., Liu C.-L., Li X.-X., Li Q.-C., Ma L.-M., Liu G.-L. (2015). VEGF Gene Polymorphisms are Associated with Risk of Tetralogy of Fallot. Med. Sci. Monit..

[38] Doi K., Noiri E., Nakao A., Fujita T., Kobayashi S., Tokunaga K. (2006). Functional Polymorphisms in the Vascular Endothelial Growth Factor Gene Are Associated with Development of End-Stage Renal Disease in Males. J. Am. Soc. Nephrol..

[39] Carmeliet P., Ferreira V., Breier G., Pollefeyt S., Kieckens L., Gertsenstein M., Fahrig M., Vandenhoeck A., Harpal K., Eberhardt C. (1996). Abnormal blood vessel development and lethality in embryos lacking a single VEGF allele. Nat. Cell Biol..

[40] Ferrara N., Carver-Moore K., Chen H., Dowd M., Lu L., O’Shea K.S., Powell-Braxton L., Hillan K.J., Moore M.W. (1996). Heterozygous embryonic lethality induced by targeted inactivation of the VEGF gene. Nat. Cell Biol..

[41] Damert A., Miquerol L., Gertsenstein M., Risau W., Nagy A. (2002). Insufficient VEGFA activity in yolk sac endoderm compromises haematopoietic and endothelial differentiation. Development.

[42] Shalaby F., Rossant J., Yamaguchi T.P., Gertsenstein M., Wu X.-F., Breitman M.L., Schuh A.C. (1995). Failure of blood-island formation and vasculogenesis in Flk-1-deficient mice. Nat. Cell Biol..

[43] Wang X., Chen D., Chen K., Jubran A., Ramirez A., Astrof S. (2017). Endothelium in the pharyngeal arches 3, 4 and 6 is derived from the second heart field. Dev. Biol..

[44] Yashiro K., Shiratori H., Hamada H. (2007). Haemodynamics determined by a genetic programme govern asymmetric development of the aortic arch. Nat. Cell Biol..

[45] Gittenberger-de Groot A.C., Azhar M., Molin D.G.M. (2006). Transforming Growth Factor [beta] -SMAD2 Signaling and Aortic Arch Development. Trends Cardiovasc. Med..

[46] Maruyama K., Miyagawa-Tomita S., Mizukami K., Matsuzaki F., Kurihara H. (2019). Isl1-expressing non-venous cell lineage contributes to cardiac lymphatic vessel development. Dev. Biol..

[47] Karkkainen M.J., Haiko P., Sainio K., Partanen J., Taipale J., Petrova T.V., Jeltsch M., Jackson D.G., Talikka M., Rauvala H. (2003). Vascular endothelial growth factor C is required for sprouting of the first lymphatic vessels from embryonic veins. Nat. Immunol..

[48] Page D.J., Miossec M.J., Williams S., Monaghan R., Fotiou E., Cordell H.J., Sutcliffe L., Topf A., Bourgey M., Bourque G. (2019). Whole Exome Sequencing Reveals the Major Genetic Contributors to Nonsyndromic Tetralogy of Fallot. Circ. Res..

[49] Dumont D.J., Jussila L., Taipale J., Lymboussaki A., Mustonen T., Pajusola K., Breitman M., Alitalo K. (1998). Cardiovascular Failure in Mouse Embryos Deficient in VEGF Receptor-3. Science.

[50] Groppa E., Brkic S., Bovo E., Reginato S., Sacchi V., Di Maggio N., Muraro M.G., Calabrese D., Heberer M., Gianni-Barrera R. (2015). VEGF dose regulates vascular stabilization through Semaphorin3A and the Neuropilin-1 + monocyte/ TGF -β1 paracrine axis. EMBO Mol. Med..

[51] Yelland T., Djordjevic S. (2016). Crystal Structure of the Neuropilin-1 MAM Domain: Completing the Neuropilin-1 Ectodomain Picture. Structure.

[52] Mehta V., Fields L., Evans I.M., Yamaji M., Pellet-Many C., Jones T., Mahmoud M., Zachary I. (2018). VEGF (Vascular Endothelial Growth Factor) Induces NRP1 (Neuropilin-1) Cleavage via ADAMs (a Disintegrin and Metalloproteinase) 9 and 10 to Generate Novel Carboxy-Terminal NRP1 Fragments That Regulate Angiogenic Signaling. Arter. Thromb. Vasc. Biol..

[53] Gu C., Rodriguez E.R., Reimert D.V., Shu T., Fritzsch B., Richards L.J., Kolodkin A.L., Ginty D.D. (2003). Neuropilin-1 conveys semaphorin and VEGF signaling during neural and cardiovascular development. Dev. Cell.

[54] Cordell H.J., Töpf A., Mamasoula C., Postma A., Bentham J., Zelenika D., Heath S., Blue G.M., Cosgrove C., Granados-Riveron J.T. (2013). Genome-wide association study identifies loci on 12q24 and 13q32 associated with Tetralogy of Fallot. Hum. Mol. Genet..

[55] Fan S.-H., Shen Z.-Y., Xiao Y.-M. (2018). Functional polymorphisms of the neuropilin 1 gene are associated with the risk of tetralogy of Fallot in a Chinese Han population. Gene.

[56] Roy S., Bag A.K., Singh R.K., Talmadge J.E., Batra S.K., Datta K. (2017). Multifaceted Role of Neuropilins in the Immune System: Potential Targets for Immunotherapy. Front. Immunol..

[57] Murphy J.F., Fitzgerald D.J. (2001). Vascular endothelial cell growth factor (VEGF) induces cyclooxygenase (COX)-dependent proliferation of endothelial cells (EC) via the VEGF-2 receptor. FASEB J..

[58] Dixelius J., Olsson A.K., Thulin Å., Lee C., Johansson I., Claesson-Welsh L. (2006). Minimal Active Domain and Mechanism of Action of the Angiogenesis Inhibitor Histidine-Rich Glycoprotein. Cancer Res..

[59] Duran I., Tenney J., Warren C.M., Sarukhanov A., Csukasi F., Skalansky M., Iruela-Arispe L., Krakow D. (2018). NRP1 haploinsufficiency predisposes to the development of Tetralogy of Fallot. Am. J. Med Genet. Part A.

[60] Koch S., van Meeteren L., Morin E., Testini C., Weström S., Björkelund H., LE Jan S., Adler J., Berger P., Claesson-Welsh L. (2014). NRP1 Presented in trans to the Endothelium Arrests VEGFR2 Endocytosis, Preventing Angiogenic Signaling and Tumor Initiation. Dev. Cell.

[61] Shaheen R., Alhashem A., Alghamdi M.H., Seidahmad M.Z., Wakil S., Dagriri K., Keavney B., Goodship J., Alyousif S., Alhabshan F. (2015). Positional mapping ofPRKD1,NRP1andPRDM1as novel candidate disease genes in truncus arteriosus. J. Med Genet..

[62] Kawasaki T., Kitsukawa T., Bekku Y., Matsuda Y., Sanbo M., Yagi T., Fujisawa H. (1999). A requirement for neuropilin-1 in embryonic vessel formation. Development.

[63] Newbern J., Zhong J., Wickramasinghe R.S., Li X., Wu Y., Samuels I., Cherosky N., Karlo J.C., O’Loughlin B., Wikenheiser J. (2008). Mouse and human phenotypes indicate a critical conserved role for ERK2 signaling in neural crest development. Proc. Natl. Acad. Sci. USA.

[64] Masuda T., Taniguchi M. (2014). Congenital diseases and semaphorin signaling: Overview to date of the evidence linking them. Congenit. Anom..

[65] van Gils J., Ramkhelawon B., Fernandes L., Stewart M.C., Guo L., Seibert T., Menezes G.B., Cara D.C., Chow C., Kinane T.B. (2013). Endothelial Expression of Guidance Cues in Vessel Wall Homeostasis Dysregulation Under Proatherosclerotic Conditions. Arter. Thromb. Vasc. Biol..

[66] Guttmann-Raviv N., Shraga-Heled N., Varshavsky A., Guimaraes-Sternberg C., Kessler O., Neufeld G. (2007). Semaphorin-3A and Semaphorin-3F Work Together to Repel Endothelial Cells and to Inhibit Their Survival by Induction of Apoptosis. J. Biol. Chem..

[67] Silversides C.K., Lionel A.C., Costain G., Merico D., Migita O., Liu B., Yuen T., Rickaby J., Thiruvahindrapuram B., Marshall C.R. (2012). Rare Copy Number Variations in Adults with Tetralogy of Fallot Implicate Novel Risk Gene Pathways. PLoS Genet..

[68] Kodo K., Shibata S., Miyagawa-Tomita S., Ong S.-G., Takahashi H., Kume T., Okano H., Matsuoka R., Yamagishi H. (2017). Regulation of Sema3c and the Interaction between Cardiac Neural Crest and Second Heart Field during Outflow Tract Development. Sci. Rep..

[69] Plein A., Calmont A., Fantin A., Denti L., Anderson N.A., Scambler P.J., Ruhrberg C. (2015). Neural crest–derived SEMA3C activates endothelial NRP1 for cardiac outflow tract septation. J. Clin. Investig..

[70] Toyofuku T., Yoshida J., Sugimoto T., Yamamoto M., Makino N., Takamatsu H., Takegahara N., Suto F., Hori M., Fujisawa H. (2008). Repulsive and attractive semaphorins cooperate to direct the navigation of cardiac neural crest cells. Dev. Biol..

[71] Brown C.B., Feiner L., Lu M.M., Li J., Ma X., Webber A.L., Jia L., Raper J.A., Epstein J.A. (2001). PlexinA2 and semaphorin signaling during cardiac neural crest development. Development.

[72] Feiner L., Webber A.L., Brown C.B., Lu M.M., Jia L., Feinstein P., Mombaerts P., Epstein J.A., Raper J.A. (2001). Targeted disruption of semaphorin 3C leads to persistent truncus arteriosus and aortic arch interruption. Development.

[73] Kodo K., Nishizawa T., Furutani M., Arai S., Yamamura E., Joo K., Takahashi T., Matsuoka R., Yamagishi H. (2009). GATA6 mutations cause human cardiac outflow tract defects by disrupting semaphorin-plexin signaling. Proc. Natl. Acad. Sci. USA.

[74] Gu C., Yoshida Y., Livet J., Reimert D.V., Mann F., Merte J., Henderson C.E., Jessell T.M., Kolodkin A.L., Ginty D.D. (2005). Semaphorin 3E and Plexin-D1 Control Vascular Pattern Independently of Neuropilins. Science.

[75] Aghajanian H., Choi C., Ho V.C., Gupta M., Singh M., Epstein J.A. (2014). Semaphorin 3d and Semaphorin 3e Direct Endothelial Motility through Distinct Molecular Signaling Pathways. J. Biol. Chem..

[76] Gitler A.D., Lu M.M., A Epstein J. (2004). PlexinD1 and Semaphorin Signaling Are Required in Endothelial Cells for Cardiovascular Development. Dev. Cell.

[77] Ta-Shma A., Pierri C.L., Stepensky P., Shaag A., Zenvirt S., Elpeleg O., Rein A.J. (2013). Isolated truncus arteriosus associated with a mutation in the plexin-D1 gene. Am. J. Med Genet. Part A.

[78] Moriya J., Minamino T., Tateno K., Okada S., Uemura A., Shimizu I., Yokoyama M., Nojima A., Okada M., Koga H. (2010). Inhibition of Semaphorin As a Novel Strategy for Therapeutic Angiogenesis. Circ. Res..

[79] Mehta V., Pang K.-L., Rozbesky D., Nather K., Keen A., Lachowski D., Kong Y., Karia D., Ameismeier M., Huang J. (2020). The guidance receptor plexin D1 is a mechanosensor in endothelial cells. Nat. Cell Biol..

[80] Chen H., He Z., Bagri A., Tessier-Lavigne M. (1998). Semaphorin–Neuropilin Interactions Underlying Sympathetic Axon Responses to Class III Semaphorins. Neuron.

[81] Takahashi T., Fournier A., Nakamura F., Wang L.-H., Murakami Y., Kalb R.G., Fujisawa H., Strittmatter S.M. (1999). Plexin-Neuropilin-1 Complexes Form Functional Semaphorin-3A Receptors. Cell.

[82] Manshaei R., Merico D., Reuter M.S., Engchuan W., Mojarad B.A., Chaturvedi R., Heung T., Pellecchia G., Zarrei M., Nalpathamkalam T. (2020). Genes and Pathways Implicated in Tetralogy of Fallot Revealed by Ultra-Rare Variant Burden Analysis in 231 Genome Sequences. Front. Genet..

[83] Hsu J.J., Vedula V., Baek K.I., Chen C., Chen J., Chou M.I., Lam J., Subhedar S., Wang J., Ding Y. (2019). Contractile and hemodynamic forces coordinate Notch1b-mediated outflow tract valve formation. JCI Insight.

